# AFFECTIVE RESPONSES TO DAILY MEMORY LAPSES IMPACT PSYCHOLOGICAL WELL-BEING IN MIDUS

**DOI:** 10.1093/geroni/igad104.1766

**Published:** 2023-12-21

**Authors:** Jennifer Turner, Jacqueline Mogle, Nikki Hill, David Almeida

**Affiliations:** University of Hawaii at Hilo, Hilo, Hawaii, United States; Clemson University, Clemson, South Carolina, United States; The Pennsylvania State University, University Park, Pennsylvania, United States; The Pennsylvania State University, State College, Pennsylvania, United States

## Abstract

Daily experiences have broad and far-reaching impacts on physical and mental health. Past work has linked micro-level experiences (e.g., daily stress) to macro-level processes (e.g., anxiety and depression; Charles et al., 2013) with a goal of understanding how day-to-day experiences influence development of physiological and psychological pathology. One mechanistic pathway for the link between daily experiences and longer-term outcomes are affective responses. This study extends previous work by examining mediation effects of positive and negative affect to a common daily experience: daily memory lapses on psychological well-being in a national sample of participants (N = 2,018; 56.6% Women, Mage = 56.92, SD = 13.37) from the MIDUS Refresher (2011-2014) and the MIDUS-3 (2013-2019). Each day participants reported on positive and negative affect and memory lapses. At the start of each wave, a comprehensive well-being assessment included different facets of psychological well-being (e.g., autonomy, environmental mastery). Daily memory lapses were significantly associated with increased negative affect and decreased positive affect on that same day. As expected, positive affect was associated with greater psychological well-being, while negative affect was associated with an inverse relationship. Importantly, significant indirect (mediation) effects were obtained for all models. This suggests that daily memory lapses have an impact on psychological well-being through their impacts on daily affect. This work provides increased evidence for the importance of emotional responses to daily stressors (such as memory lapses) being a determinate of distal health-related outcomes.

